# Monomolecular covalent honeycomb nanosheets produced by surface-mediated polycondensation between 1,3,5-triamino benzene and benzene-1,3,5-tricarbox aldehyde on Au(111)†

**DOI:** 10.1039/d0na00180e

**Published:** 2020-05-28

**Authors:** Masashi Kunitake, Ryota Tanoue, Rintaro Higuchi, Soichiro Yoshimoto, Ryusei Haraguchi, Shinobu Uemura, Nobuo Kimizuka, Adam Z. Stieg, James K. Gimzewski

**Affiliations:** Institute of Industrial Nanomaterials, Kumamoto University 2-39-1 Kurokami, Chuo-ku Kumamoto 860-8555 Japan kunitake@kumamoto-u.ac.jp; Graduate School of Science and Technology, Kumamoto University 2-39-1 Kurokami, Chuo-ku Kumamoto 860-8555 Japan; Faculty of Engineering and Design, Kagawa University 2217-20 Hayashi-cho Takamatsu Kagawa 761-0396 Japan; Department of Chemistry and Biochemistry, Graduate School of Engineering, Center for Molecular Systems (CMS), Kyushu University 744 Moto-oka, Nishi-ku Fukuoka 819-0395 Japan; California NanoSystems Institute 570 Westwood Plaza Los Angeles CA 90095 USA; WPI Center for Materials Nanoarchitectonics (MANA), National Institute for Materials Science (NIMS) 1-1 Namiki Tsukuba Ibaraki 305-0044 Japan; Department of Chemistry and Biochemistry, University of California-Los Angeles 607 Charles E. Young Drive East Los Angeles CA 90095 USA

## Abstract

Fabrication of a two-dimensional covalent network of honeycomb nanosheets comprising small 1,3,5-triamino benzene and benzene-1,3,5-tricarboxaldehyde aromatic building blocks was conducted on Au(111) in a pH-controlled aqueous solution. *In situ* scanning tunneling microscopy revealed a large defect-free and homogeneous honeycomb π-conjugated nanosheet at the Au(111)/liquid interface. An electrochemical potential dependence indicated that the nanosheets were the result of thermodynamic self-assembly based not only on the reaction equilibrium but also on the adsorption (partition) equilibrium, which was controlled by the building block surface coverage as a function of electrode potential.

## Abstract 2

A large defect-free and homogeneous honeycomb π-conjugated nanosheet consisted of small aromatic building blocks at the solution/solid interface.

## Graphical abstract

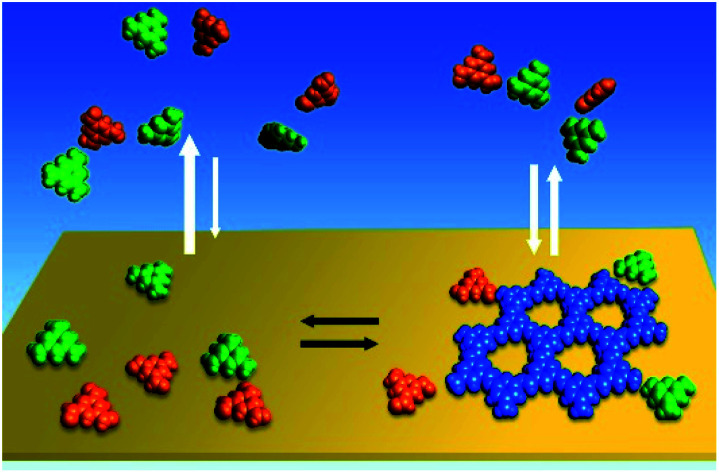

## Introduction

Among novel nanomaterials with high-dimensional regularity, such as porous coordination polymers (PCPs),^1^ metal–organic frameworks (MOFs),^2,3^ and covalent organic frameworks (COFs),^4–6^ two-dimensional (2D) covalently bonded macromolecular frameworks and nanosheets are a novel category of nanomaterials that have attracted a wealth of interdependent research.^7–13^ As typical 2D nanomaterials, graphene and graphene-derived analogues, including graphene oxide, have been widely studied for their electronic and optical properties based on two-dimensionally expanded conjugated systems.^14^ Furthermore, inorganic nanosheets fabricated from the exfoliation of layered crystals have also been synthesized.^15,16^

Conversely, on-site syntheses of 2D covalently bonded macromolecular systems on substrates, by adopting an organic synthesis and coordination chemistry strategy, have received significant interest recently. Highly organized macromolecular systems based on conjugated covalent bonds and/or metal–organic coordination are expected to produce a broad spectrum of optical and electronic properties based on the diverse and unlimited design potential of organic chemical structures.^17–20^

Under ultrahigh vacuum (UHV) conditions, the synthesis of 2D covalent systems based on reactions such as thermally initiated C–C couplings,^21–23^ Ullmann couplings between aromatic halogen-terminated molecules,^24–28^ esterification between boronic acid and hydroxyl units,^29^ and the Schiff base (also known as imine or azomethine) between primary amines and aldehyde units^13,30^ have been reported.^31^ Under UHV, the dose of the molecules (surface coverage) can be controlled as a function of evaporation rate and time, prior to thermal treatments, to induce coupling reactions. In the case of irreversible coupling reactions in UHV, the construction of covalent molecular systems at relatively low surface coverages would be a typical strategy to avoid irregular reactions. Typically, adlayers are frequently subjected to heat treatment under UHV conditions to improve the regularity of ordered structures by enhancing the reversibility of the reaction. The acceleration of the coupling–decoupling exchange reaction between the component molecules on the thermodynamic equilibrium is key to establishing an ideal ordered structure with a high degree of symmetry and few defects.

At vapor/solid interfaces, the on-site fabrication of 2D macromolecular frameworks, such as boronic acid COF^32^ and surface mounted metal–organic frameworks^33^ has been achieved by mild thermal treatments in the presence of water vapor^32^ or an acetic acid vapor environment.^33^ By-product formation during reversible reactions accelerates the recombination of the coupling bonds between components by decreasing the activation energy, leading to Ostwald ripening. Furthermore, at solid/liquid interfaces, the self-assembly of 2D covalent macromolecular frameworks has been achieved by delicate and simultaneous management of the adsorption equilibrium and the coupling–decoupling reaction equilibrium. Previous reports have focused on the thermodynamic self-assembly of 2D covalent frameworks based on aromatic Schiff base (azomethine) coupling reactions.^34–37^ The high reactivity and reversibility of the coupling reactions enable surface-mediated selective polycondensation and thermodynamic self-assembly in an ambient aqueous solution at room temperature, in a similar manner to the non-covalent systems based on hydrogen bonds^38,39^ and halogen bonds.^40–42^ The arrays of π-conjugated linear polymers, porphyrin-based 2D mesh frameworks, and supramolecular arrangements of distinct molecular systems have been fabricated on iodine-modified Au(111) (I/Au(111)).^34^ Additionally, continuous 3D film growth of macromolecular systems (chemical liquid deposition) beyond the formation of 2D polymeric architectures has been reported.^43,44^

**Scheme 1:**
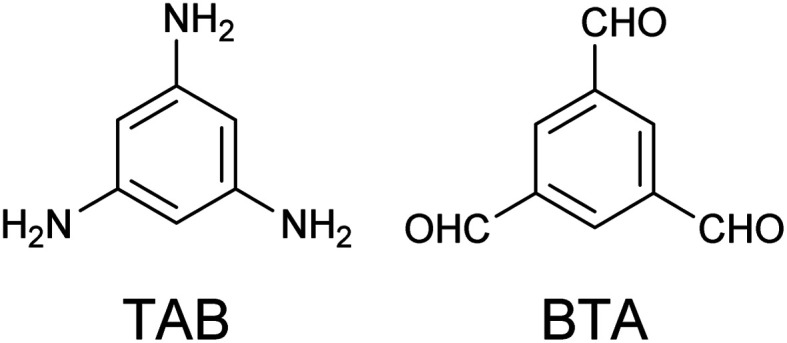
Chemical structures of 1,3,5-triamino benzene (TAB, left) and benzene-1,3,5-tricarbox aldehyde (BTA, right).

Herein, a homogeneous 2D honeycomb π-conjugated nanosheet comprising 1,3,5-triamino benzene (TAB) and benzene-1,3,5-tricarboxaldehyde (BTA) (Scheme 1) aromatic building blocks assembled at an Au(111)/liquid interface is reported. Lei and co-workers have reported the construction of 2D-COFs by a combination of aromatic diamine and BTA at the octanoic acid/single-layer graphene interface on copper foil.^45,46^

## Experimental

### Materials

TAB (MER Co. Ltd., 99.95%), BTA (MER Co. Ltd., 98%), NaClO_4_ (MER Co. Ltd., 98%), and HClO_4_ (Nacalai Tesque, 70%) were used without further purification

### Typical preparation conditions

A 0.1 M aqueous NaClO_4_ solution containing 0.1 mM TAB and 0.1 mM BTA was prepared with ultrapure water purified by a Milli-Q system (Millipore). A pH between 3 and 5 was controlled by the addition of 0.1 mM HClO_4_. A well-defined bare Au(111) single-crystal bead was prepared by flaming and quenching. I/Au(111) surfaces were prepared by immersing the Au(111) substrates into a 10 mM KI aqueous solution, followed by rinsing with ultrapure water.

### Electrochemical scanning tunneling microscopy (EC-STM)

The details of the *in situ* STM investigations have been previously reported.^39^ The EC-STM measurements were performed with a Nanoscope E (Digital Instruments, Santa Barbara, California), with an electrochemically etched tungsten tunneling tip that was sealed with transparent nail polish. All images were collected in a constant current mode. Two Pt wires were used as quasi-reference and counter electrodes in the STM cell, respectively. The measured potentials were calibrated with the saturated calomel electrode (SCE) or Ag/AgCl reference electrode after the measurements were taken. All potentials were reported with respect to a reference hydrogen electrode (RHE).

## Results and discussion

The symmetrical benzene analogues, TAB and BTA, which have three primary amino or aldehyde groups in a three-fold symmetrical position, respectively, were selected as a new combination for the Schiff base coupling reaction. Deriving from a covalent bond framework structure from the combination of TAB and BTA, an alternate 2D connected honeycomb structure is expected to be fabricated. Eventually, 2D covalent honeycomb nanosheets on an Au(111) surface were successfully fabricated *via* the spontaneous adsorption and on-site polycondensation of the building blocks from an aqueous solution at room temperature (between 20 and 25 °C).

To self-assemble highly-ordered covalently bonded systems, both the adsorption–desorption and coupling–decoupling reaction equilibria must be carefully controlled at the interface. Adequate physical adsorption strength is a pre-requisite for adsorption-induced self-assembly. “Too strong” adsorption prohibits surface diffusion for self-assembly and “too weak” adsorption provides insufficient numbers of surface molecules. The majority of the solution conditions, such as individual component concentrations and temperature, are critical parameters that manage the thermodynamics of the adsorption and reaction equilibria. Hence, each factor must be optimized for each system containing different building blocks and substrates.

Conversely, the reaction equilibrium of Schiff base coupling is primarily controlled by the solution pH, although other parameters, such as concentration and temperature, also have an influence. Under basic pH conditions, the reaction readily proceeds and frequently forms irreversible sedimentation of polycondensation products. At a pH below the p*K*_a_ of the aromatic amines, the coupling equilibrium shifts to “breakdown” as a result of the protonation of the primary amino groups. A particular point to note is the promotion of the dehydration reaction on a hydrophobic surface in contrast to that in a bulk solution. The suppressing coupling reaction in an aqueous solution phase together with the promotion of the selective dehydration reaction onto a hydrophobic solid substrate are the crucial factors for adsorption-induced covalent self-assembly.

The typical optimum conditions for the fabrication of 2D honeycomb nanosheets on Au(111) are: 0.1 M NaClO_4_ solution, 0.1 mM TAB and 0.1 mM BTA at pH 4.5. Under such conditions, the solution was observed to be homogeneous with no precipitate (Fig. S1†), indicating a dominant and reversible polycondensation at the surface only.

We explored the conditions for self-assembly widely in terms of the concentrations of the samples from 0.01 mM to the saturation and the salt from 10 mM to nearly saturation, and the pH range between 2 to 8. At high acidic conditions pH < 3, an intact Au(111) structure was observed instead of molecular images. It is due to the hindrance of the coupling reaction by TAB protonation. At the higher pH over 6, unclear images were mainly observed although the honeycomb structure was observed a few times. The excess adsorption or formation of multilayers may be concerned. In the lower concentration of less than 0.01 mM of the samples, and/or less than 0.01 M of NaClO_4_, we could not find any molecular structure, probably due to a low surface partition of TAB and BTA. In the case of the higher concentration up to roughly 0.5 mM of the sample or 1 M of NaClO_4_, the same honeycomb structure was formed. The reduction of the temperature may compensate for low reagent concentration conditions for the covalent self-assembly, but we could not conduct the experiments for temperature dependence due to the experimental limitation. Additionally, no adlayer was observed in the solutions in the presence of individual component BTA or TAB for all solution conditions we applied.

As previously mentioned, the molecule–substrate interaction (adsorption strength) is also a crucial factor to manage the thermodynamic self-assembly in terms of adsorption strength. The relationships between self-assembly and adsorption strength for various single-crystal metal substrates have been summarized previously.^47^ In a preliminary experiment, the self-assembly of TAB and BTA on I/Au(111), under similar solution conditions, was also examined because porphyrin-based covalent 2D mesh structures have been successfully fabricated on I/Au(111).^34^ However, no molecular image was observed at all on I/Au(111) under the conditions including high reactant concentrations (nearly saturated concentration) and/or the pH up to 8. These observations are as a result of the weak interactions of small molecules, such as TAB and BTA, against I/Au(111), unlike stronger interactions observed with relatively larger molecules, such as porphyrins. Additionally, similar small molecules, such as trimesic acid (TMA) and melamine, have formed honeycomb network structures on Au(111) in a solution.^48,49^ In the systems, the complementary hydrogen bonds are strongly stabilized the systems. In another word, strong intermolecular interactions are necessary for the self-assembly of small molecules at the interface.

Iodine adlayers on Au(111) are known to be easily removed in an electrolyte solution by electrochemical reduction at a negative potential.^50^ Typically, the fabrication of the honeycomb nanosheet on Au(111) first involves the introduction of I/Au(111) into the cell containing the solution in the presence of TAB and BTA. Thereafter, an intact Au(111) surface was exposed by electrochemical reduction to induce the self-assembly resulting in the spontaneous formation of honeycomb sheets on the Au(111) surface at the near open-circuit potential (OCP) region (approximately +0.8 V *vs.* RHE).

**Fig. 1:**
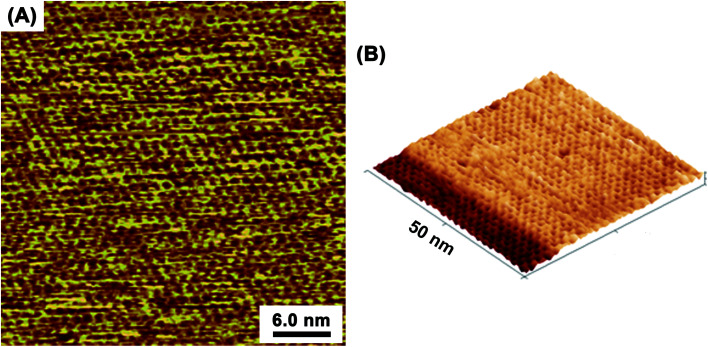
*In situ* scanning tunneling microscopy (STM) images ((A) topographic image; (B) 3D image) of a TAB–BTA honeycomb network nanosheet on Au(111). The images were captured at open-circuit potential in a solution containing TAB and BTA. Honeycomb structures fabricated on upper and lower atomic terraces including Au single atomic steps are shown.

At the optimized solution conditions, highly-ordered adlayers comprising TAB–BTA, featuring a honeycomb lattice structure completely covering the atomically flat Au(111) terraces, were observed by *in situ* EC-STM. Fig. 1A and B show wide-area *in situ* STM images of the TAB–BTA honeycomb adlayers self-assembled on Au(111). In Fig. 1B, the honeycomb lattice is observed to completely cover an Au(111) terrace up to the step edge of a lower terrace.

**Fig. 2:**
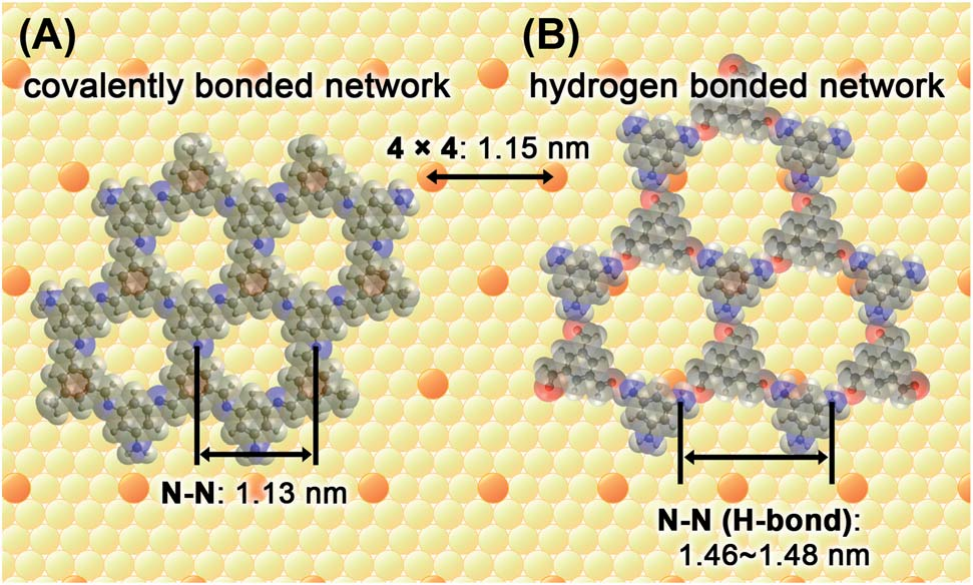
Comparison of honeycomb models on Au(111) for covalent bond (A) and hydrogen-bond (B) networks. The blue and red spots in the network models, and orange spots on the Au(111) lattice, indicate the positions of nitrogen and oxygen atoms, and the (4 × 4) position, respectively.

Fig. 2 shows two 2D honeycomb models, a covalent bonded framework (A) and a hydrogen-bond network (B), to evaluate the honeycomb structure observed by STM imaging. In the latter model, hydrogen bonds are assumed to bridge between the hydrogen atoms on the primary amino groups of TAB and the oxygen atoms on the aldehyde groups of BTA. By comparing the STM images and both the models, the observed lattice parameters correspond to the expected shapes and sizes of a lattice that comprises alternatively connected TAB and BTA molecules *via* covalent bonds. Theoretical calculations were performed for the covalent honeycomb model and the hydrogen-bonded honeycomb model, which yielded honeycomb lattice distances (N–N distance) of 1.13 and 1.46–1.48 nm, respectively. The unit cell distance of the hexagonal shape observed from STM, *ca.* 1.2 nm, is approximately equal to the value calculated from the covalent bond model. Furthermore, the theoretical and experimental distances for the covalent honeycomb are similar to the repeating distance of Au(111)-(4 × 4), 1.152 nm. The orientations of the honeycomb lattice, observed from experimental data, matched with those of the underlying atomic lattice of Au(111), which demonstrate that the honeycomb lattice was commensurate with the Au(111)-(4 × 4) substrate.

The combination of trifunctional BTA and bifunctional DAB (1,4-diaminobenzene)^45,46^ was also preliminarily examined for covalent-self-assembly in solution. In the system of BTA and DAB, incomplete honeycomb nanosheets with a large cavity, that corresponded to the expected model, were formed as shown in Fig. S2.† During the observation, the incomplete honeycomb structure was maintained, but the local structure such as defect positions was always changed. Although an incomplete honeycomb, formation of the large honeycomb consistent with the expected size depending on the combination of building block molecules and the dynamics of large honeycomb strongly suggest that both TAB/BTA and BTA/DAB systems are covalent networks.

In contrast to the BTA/DAB system and porphyrin-based covalent 2D meshes on I/Au(111),^34^ the honeycomb adlayers of the TAB/BTA system on Au(111) were extremely uniform with few defects and boundaries. The reasons for the high degree of uniformity are: (1) the small size of the honeycomb cavity produced by the small building blocks; (2) the high structural symmetry in the likely absence of irregular defects; (3) the relatively fast exchange reactions of the coupling–decoupling equilibrium; and (4) the relatively simple epitaxy against the substrate lattice.

**Fig. 3:**
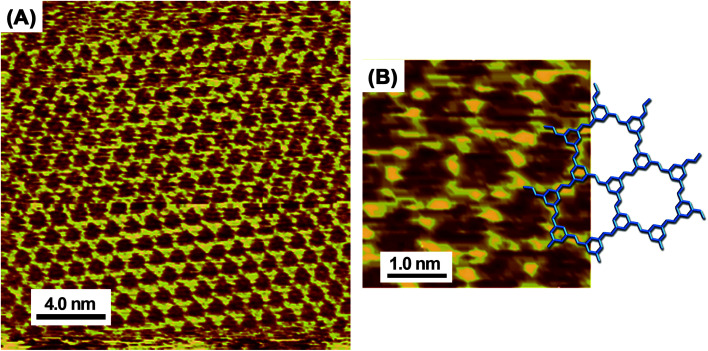
High-resolution *in situ* STM images (A and B) of TAB–BTA honeycomb network nanosheets on Au(111) and the corresponding chemical model that is overlapped on the image (B).

Fig. 3 shows sub-molecular high-resolution STM images of the honeycomb network that reveal an architecture consisting of flat-oriented TAB and BTA molecules. The model overlapped on the STM image (Fig. 3B) shows that the honeycomb features correspond to the expected shapes and sizes of a lattice where TAB and BTA molecules are alternately connected *via* covalent bonds. Furthermore, the existence of a honeycomb surface chirality depends on the orientation of the CH=N bond, which is also complementary to the chemical model. However, the limited resolution prevented the distinction between the TAB and BTA benzene units and/or the anisotropic locations of the nitrogen atoms in the azomethine double bonds (Fig. S3†). Additionally, almost no domain boundaries were observed, and the possibility of mixing right-handed and left-handed domains could not be proved.

For thermodynamic self-assembly at a solid/liquid interface, the electrode potential is a crucial parameter to regulate the adsorbate–substrate interactions that induce self-organization on the surface. Typically, the surface coverage of neutral adsorbates in a solution is known to decrease as a function of deviations of electrode potential from OCP, because of the increased negative or positive surface charges.^51^ A negatively polarized surface can disturb the adsorption of neutral molecules because of the electrostatic interactions of counter-ions approaching the surface. In the case of reversible physisorption, the surface coverage of the building blocks can be precisely controlled as a function of the electrode potential at the mV level. Thus, “order–disorder” or “order–order” phase transitions induced by the electrode potential management have been demonstrated in aqueous solution systems using EC-STM.

Though the honeycomb nanosheets on Au(111) were successfully fabricated with no potential control (nearly OCP), the destruction and reconstruction of the honeycomb nanosheets were also achieved by controlling the electrode potential. The honeycomb nanosheets were observed at a potential region of 0.3–0.8 V *vs.* RHE. When the potential was reduced to <0.3 V *vs.* RHE, the honeycomb nanosheets were no longer observed, indicating a collapse of the covalent system. Conversely, at an excessive positive potential, unclear and irregular structures, which may be surface reaction products as a result of uncontrolled coupling, were observed at ∼0.9 V.

**Fig. 4:**
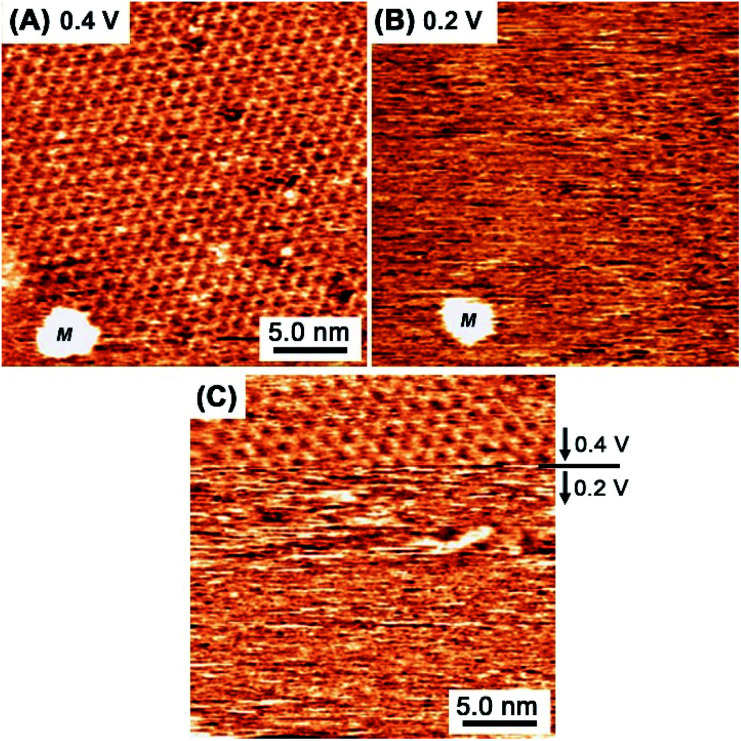
*In situ* STM images obtained at 0.4 V (A) and 0.2 V (B) in a mixed aqueous solution comprising TAB and BTA. (A) and (B) were captured at the same location on the Au(111) surface, where “M” is an aggregated island used as a fiducial marker. During the scanning in (C), the potential was abruptly changed from 0.4 V to 0.2 V at the marked line.

Fig. 4A and B show *in situ* STM images captured at essentially the same location at 0.4 V (A) and 0.2 V (B) in a mixed aqueous solution of TAB and BTA. When the potential was reduced from 0.4 V to 0.2 V, the honeycomb structure was no longer observed, indicating the collapse of the covalent bond network. At lower potentials near hydrogen evolution at <+0.1 V, the molecules completely desorbed and an intact reconstructed Au(111) surface appeared exhibiting typical double corrugation lines. The formation and collapse of the honeycomb structure at 0.4 V and 0.2 V, were reversibly controlled by the potential, respectively. In Fig. 4C, when the potential was abruptly changed from 0.4 V to 0.2 V during scanning, the honeycomb network instantly disappeared. Immediately after changing the potential to 0.2 V, incomplete oligomeric products were observed as obscure features as a result of the dynamic reaction and rapid surface diffusion, indicating rapid destruction within several seconds.

**Fig. 5:**
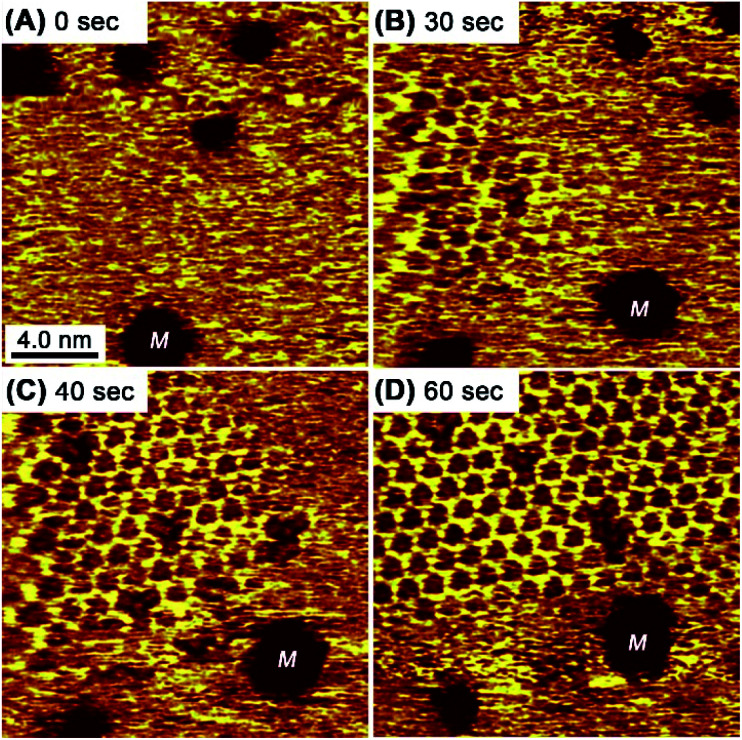
Consecutive STM images of the honeycomb mesh after the Au(111) substrate potential was changed from 0.3 V to 0.4 V at 0 s. The etch pits indicated with “M” are fiducial markers.

In contrast to the rapid collapse, the restoration of the honeycomb structure typically requires 60 s. Fig. 5 shows consecutive STM images of the honeycomb reconstruction after the substrate potential was abruptly changed from 0.3 V to 0.4 V. The reconstruction process involved the formation and growth of a honeycomb island (an intentionally produced etch pit “M” was used as a position marker in Fig. 5). The rarely observed defects in the honeycomb lattice from the standard preparations were observed here because of the electrochemically induced rapid rearrangement. The defect positions of the honeycomb changed, indicating recombination at the edges. Eventually, the defects were no longer observed as a result of Ostwald ripening at a constant potential.

The disappearance of the honeycomb frameworks at negative potentials indicates that covalent nanostructures were also subject to delicate thermodynamic equilibrium balances similar to hydrogen-bond-based thermodynamic self-assembly systems. The electrode potential dependence for the covalently bonded honeycomb structure was similar to those of the hydrogen-bonded self-assembly of TMA^48^ and melem (2,5,8-triamino-tri-*s*-triazine)^49,52^ on Au(111). In either the TMA or melem case, the electrode potential-induced “order–order” phase transitions between a hydrogen-bond-driven honeycomb structure and a density-driven closed-packed structure were observed. However, in the TAB–BTA mixed system, the honeycomb was only observed as an ordered structure with no “order–order” phase transitions observed, indicating no other metastable structure. During cyclic voltammetry measurements that compared the presence or absence of TAB and BTA, there was no clear difference corresponding to an “order–disorder” transition (Fig. S4†).

**Fig. 6:**
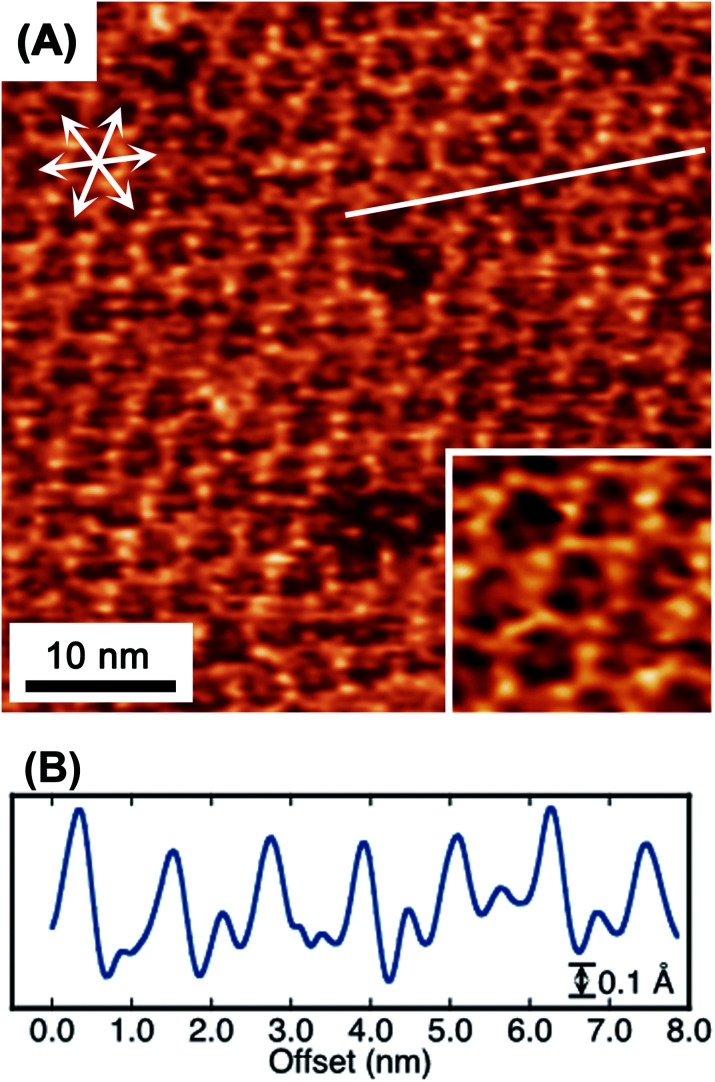
Molecular-resolution *in situ* STM image (A) of a TAB–BTA honeycomb mesh on Au(111). The inset in (A) is a higher magnification image of (A). A line scan (B) along the solid line in (A).

When the potential was cycled to induce the collapse and reconstruction, a honeycomb framework, having a unique inside structure, was unintentionally observed, as shown in Fig. 6. Within each gap of the honeycomb, an underlying honeycomb structure was observed with an apparent shift in location, indicating the possibility to form a honeycomb with a double-layered structure. Alternatively, the features may correspond to a guest molecule trapped in the honeycomb. Interestingly, the apparently lower protrusion height in the honeycomb, compared with the honeycomb framework height, would exclude the potential of the imaging of the trapped TAB or BTA molecules. In porphyrin-based covalent frameworks, the building block molecules are frequently observed as trapped guests within the mesh framework.^39^ Although there is a slight possibility that the trapped species in the frameworks were ions or water molecules, the guest candidates appear to be too small to be trapped in the cavity. Although elucidation of this suggestion is not certain, the image is thought to reveal that the multilayer film structure does not include the said trapped guest molecules.

The honeycomb structure was observed after exposure to air (Fig. S5†). Unlike under UHV conditions and in the electrolyte solution, the STM imaging noise level is significantly higher when in air. However, regardless of the noise level the image obviously proved that the honeycomb structure was maintained even when taken out of the solution and subjected to the gas phase, despite being easily broken electrochemically.

## Conclusion

In summary, covalently bonded honeycomb nanosheets were formed *via* surface-mediated polycondensation in an aqueous solution and the resultant products imaged by *in situ* STM. In contrast to dry processes under UHV, these low-cost and eco-friendly solution methods were performed with simple equipment under mild conditions. The honeycomb framework, in which TAB and BTA rings are alternatively connected to each other *via* CH=N bonds in a honeycomb structure, possesses a 2D expanded π-conjugated system that is expected to demonstrate potentially useful electronic, optical and catalytic properties.

## Conflicts of interest

The authors declare no competing interests.

## Supplementary Material

NA-002-D0NA00180E-s001
