# Supplementary material for: Monomolecular covalent honeycomb nanosheets produced by surface-mediated polycondensation between 1,3,5-triamino benzene and benzene-1,3,5-tricarbox aldehyde on Au(111)
Source: Nanoscale Adv. 2020 May 28;2(8):3202–8. doi: 10.1039/d0na00180e (PMC9417909; doi:10.1039/d0na00180e)
Supplement: NA-002-D0NA00180E-s001 [file NA-002-D0NA00180E-s001.pdf]

Electronic Supplementary Material (ESI) for Nanoscale Advances.
This journal is © The Royal Society of Chemistry 2020

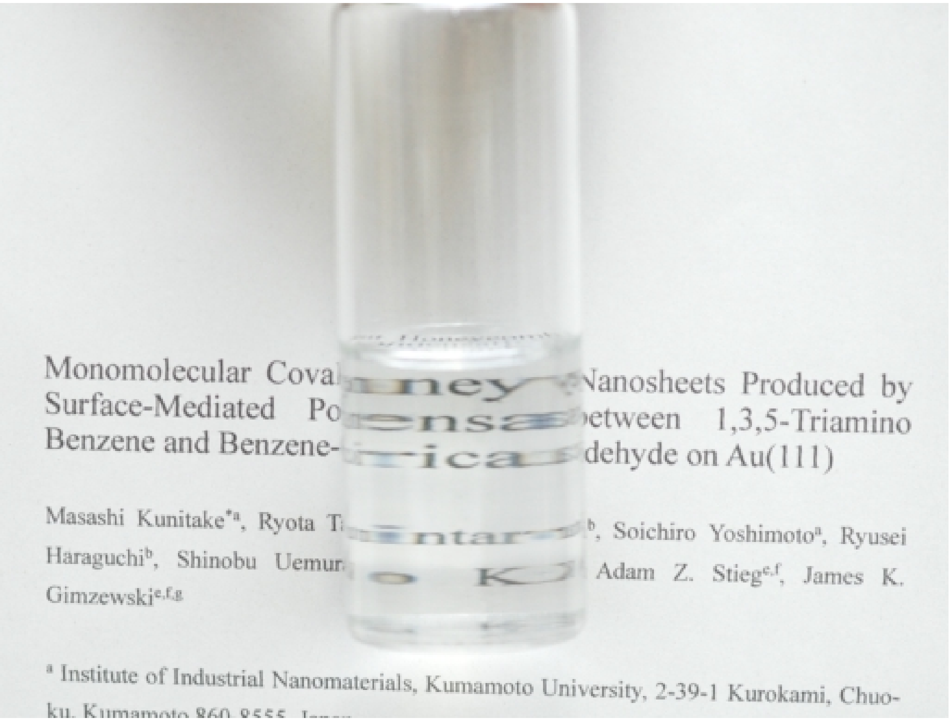

Figure S1. The photo of 0.1 M $NaClO_4$ aqueous solution in presence of 0.1 mM TAB and 0.1 mM BTA at pH 4.5

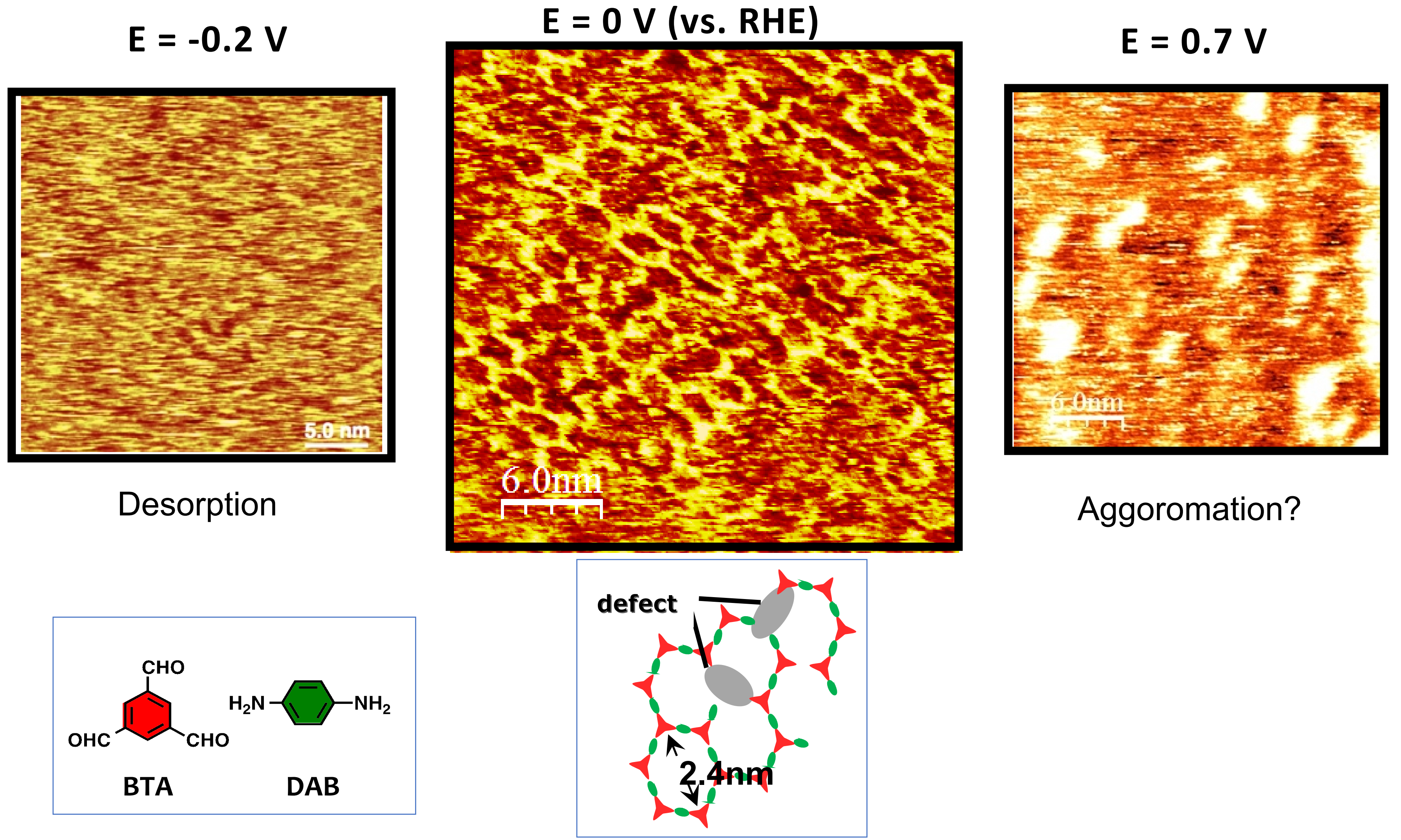

Figure S2. *In situ* STM images of incomplete honeycomb nanosheets constructed on Au(111) in 0.1 M $NaClO_4$ in the presence of 0.1 mM BTA and 0.1 mM DAB (1,4-diaminobenzene) at pH 3.8. Incomplete honeycomb nanosheets with a large cavity which corresponded to the expected model were observed at 0 V vs. RHE. During the observation, the incomplete honeycomb structure was maintained, but the local structure such as defect positions was always changed. At -0.2V, no molecular image was observed. Unclear protrusions in several nm scales appeared at 0.7 V and those were expected as the product of uncontrol reaction or aggregation.

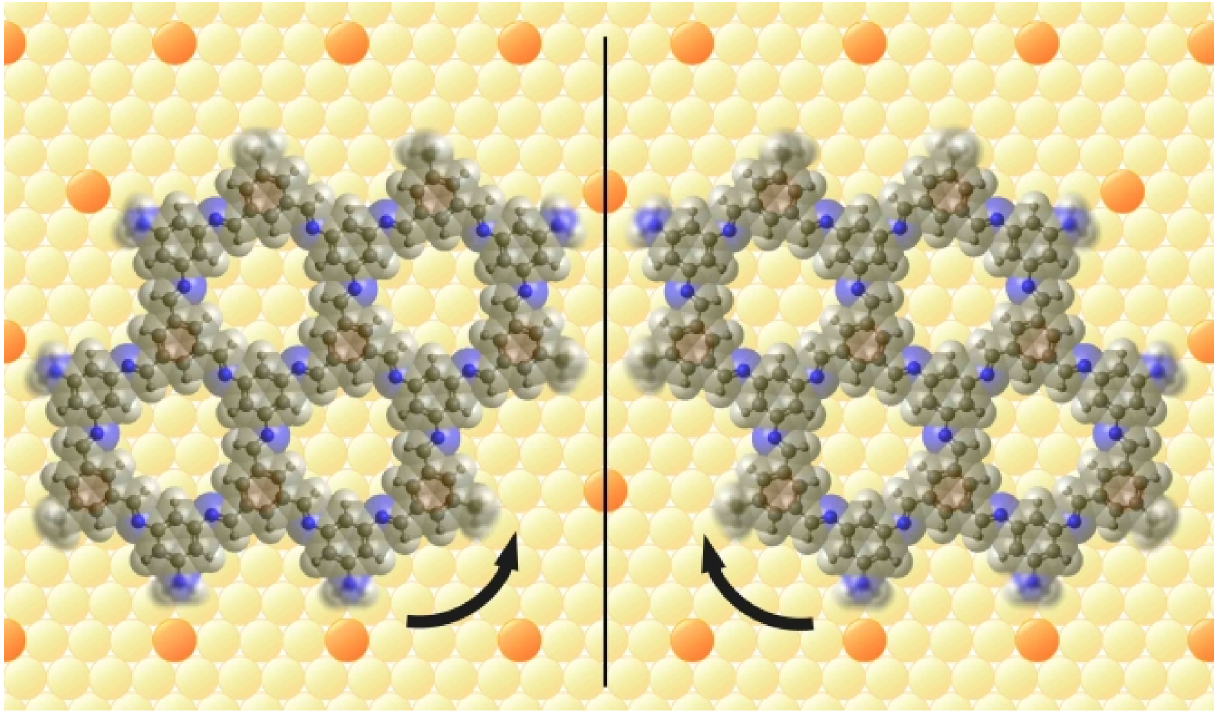

Figure S3. Tentative models of chiral 2D covalent-bonded structures.

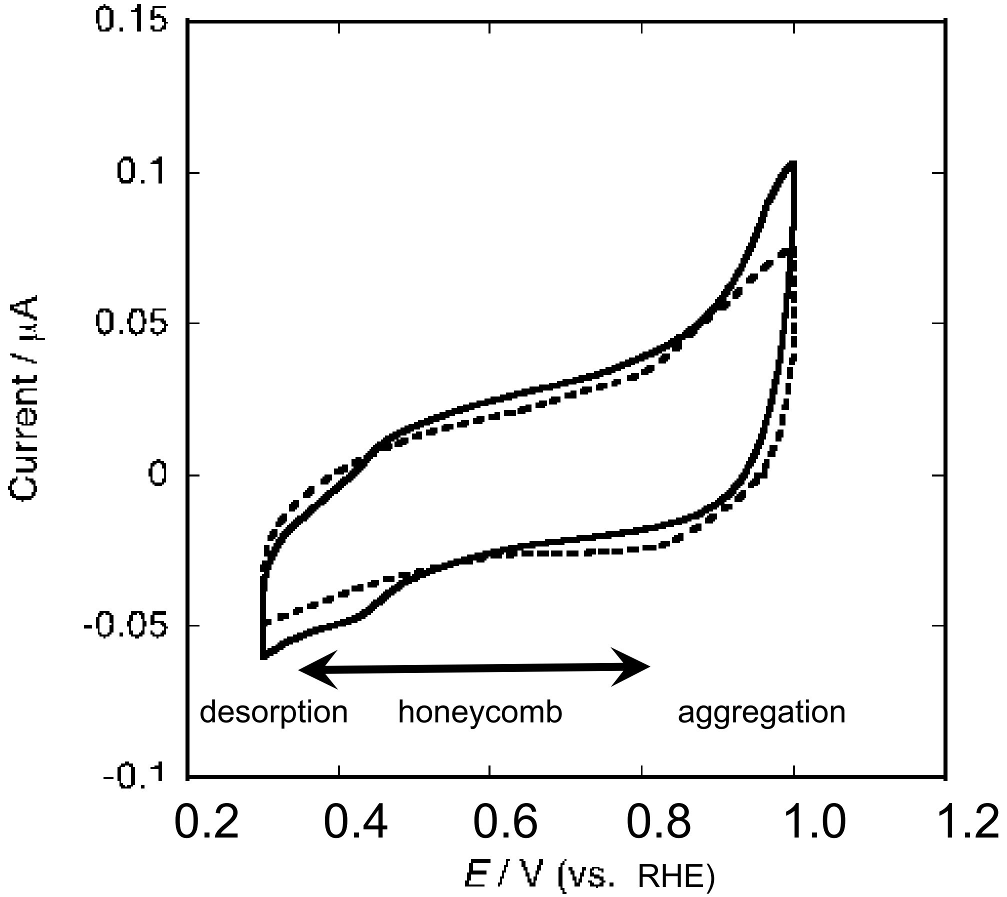

Figure S4. Cyclic voltammograms of Au(111) electrodes in 0.1 M $NaClO_4$ in absence (dotted line) and in presence (solid line) of 0.1 mM TAB and 0.1 mM BTA.

Experimental:

Well-defined bare Au(111) electrodes which were prepared by cutting and mechanically polishing the Au single crystal bead, were used for voltammetric measurements, as described in previous papers. The Au(111) electrodes were annealed in a H_2 flame and quenched in pure water saturated with H_2. The electrodes were transferred into an electrochemical cell filled with the solution as same as the solution for STM measurements.

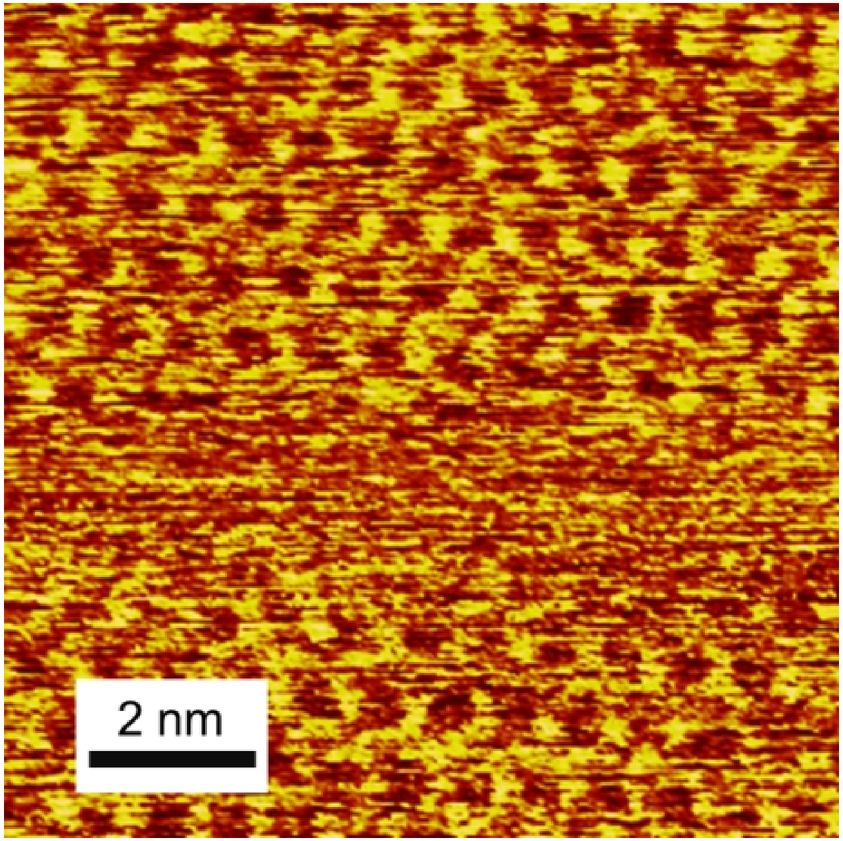

Figure S5. STM image of a TAB–BTA honeycomb structure on Au(111), which was collected once the honeycomb structure was subjected to air after removing from the solution.
